# Study on the changes during the fermentation of the wine prepared from palm (*Phoenix sylvestris*) sap

**DOI:** 10.1016/j.heliyon.2024.e35799

**Published:** 2024-08-05

**Authors:** Nabin Khadka, Dev Raj Acharya, Anish Dangal, Kishor Rai, Gaurav Gurung, Girija Sherma, Sabin Bahadur Khatri, Navin Gautam

**Affiliations:** a Central Department of Food Technology, Tribhuvan University, Dharan, 56700, Nepal; b Department of Food Technology, Nilgiri College, Tribhuvan University, Itahari, 56705, Nepal; c Department of Management, Mahendra Multiple Campus, Tribhuvan University, Dharan, 56700, Nepal; d Department of Food Technology, Central Campus of Technology, Tribhuvan University, Dharan, 56700, Nepal

**Keywords:** Palm sap, Wine, *Toddy*, Fermentation, Physico-chemical analysis, Sensory analysis

## Abstract

The sugary sap of different palm trees is fermented to create palm wine, an alcoholic beverage. This work was aimed at studying the changes that occur during the fermentation process of wine made from the sap of the wild date palm species *Phoenix sylvestris*. At first, the best age of the palm tree was determined by observing total soluble solid and sap yield for 24 h and was found to be middle-aged palm plants (15–40 years old). Pure wine yeast (*Saccharomyces cerevisiae* SC22) and a natural starter culture were added to the palm saps, adjusting the total soluble solid (TSS) to 21.5° brix (°Bx). Total titratable acidity, pH, volatile acidity, reducing sugar, non-reducing sugar, total sugar, alcohol content, ester content, and aldehyde contents were the parameters under investigation. The statistical analysis showed significant (p ≤ 0.05) changes in the physico-chemical and volatile constituents of palm sap during the fermentation process in both systems. Sensory evaluation revealed that palm wine fermented with pure yeast culture was significantly superior to natural, spontaneously fermented wine. The acceptability test showed that the ideal characteristics of palm wine are cloudy in appearance, fruity in aroma, and sweet in taste.

## Graphical abstract

**Figure:**
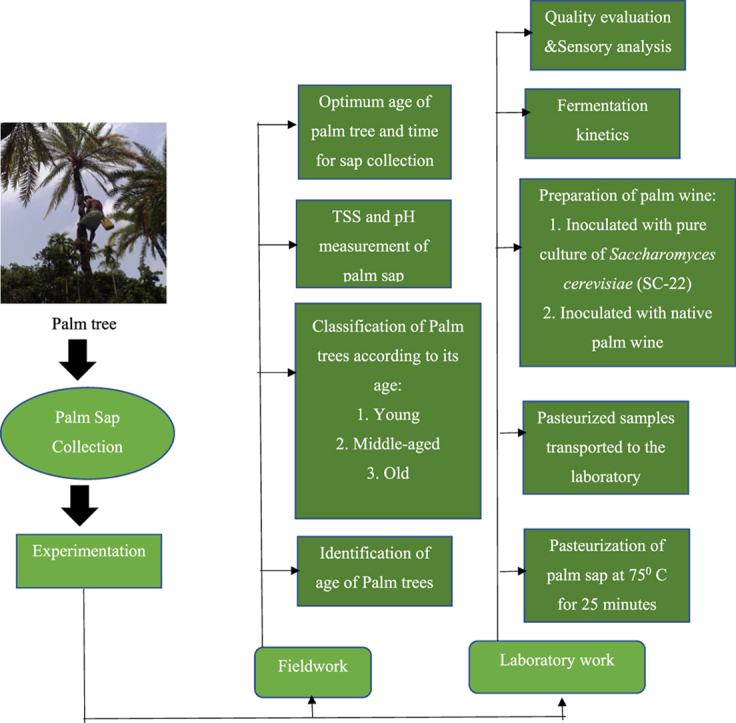

## 1 Introduction

Palm wine, also known as *Toddy* [1], is an alcoholic beverage prepared by fermenting sugary sap from various palm plants, resulting in flavors ranging from sweet and unfermented to sour, vinegary, and fermented [2]. It is known as a traditional beverage that is prepared by collecting sap from palm trees and is enjoyed in many regions across the globe [3].

Palm juice/sap is rich in minerals such as sodium (Na), potassium (K), calcium (Ca), and iron (Fe), along with ascorbic acid and polyphenols [4]. Due to its antioxidant properties, it can be considered a nutritious and healthy beverage [5]. Beverages and the sap of palm have been traditionally utilized as folk medicine, offering several medical advantages like enhanced vision and gastrointestinal health [6]. Saps obtained from the inflorescence of different palm tree species are abundant in both sugar and minerals, creating favorable conditions for the proliferation of yeast and lactic acid bacteria (LAB). Fermentation begins shortly after the collection of sap, and within a few hours, the alcohol content of the sap reaches up to 4–5% [7,8].

*Saccharomyces cerevisiae* [9] and *Zymomonas mobilis* [10] are the main contributors to the production of alcohol in palm. *Saccharomyces cerevisiae* is naturally colonized in palm sap for spontaneous fermentation to convert sugary sap to ethanol, lactic acid and other volatiles [11,12]. Palm wine contains probiotic organisms such as *Bacillus, Saccharomyces, Lactobacillus, Streptococcus,* and *Leuconostoc.* These probiotics play a role in producing vitamins, digestive enzymes, and enhancing the immune system [2]. In rural India and in the Terai region of Nepal, palm sap and wine are consumed for their specific health benefits like eyesight and gastrointestinal health improvement [13,14].

According to previous studies [[1], [2], [3],^11^,^12^], despite the high content of vitamins and minerals, in palm sap, it has been experienced a decline in market share due to the variability in its quality. To enhance consistency and market appeal, it is essential to optimize and standardize the conditions for fermentation [[15], [16], [17]]. Palm sap serves multiple purposes, not only as a valuable source of sugars and alcoholic beverages but also as an acidulant. A study of the changes occurring during the fermentation process can offer insights into adjusting the fermentation conditions to align with specific end-use requirements [18].

Traditional palm wine fermentations are typically unregulated labor-intensive processes, perceived as primitive, and often lacking in hygiene standards. These methods are typically not integrated into the formal economy, making them challenging to tax. Additionally, their potential for export is limited, and in certain cases, questions arise regarding their impact on nutritional value and safety [19]. In the traditional process of palm wine fermentation, all the steps are carried out without any control over factors such as initial solid content, temperature, and pH. This method depends on mixed culture and spontaneous fermentation, resulting in inconsistent product quality. A number of challenges are associated with various aspects of palm wine (*Toddy*) fermentation processes. This study places a significant emphasis on the effective utilization of palm sap through fermentation. The key objective of this work was to study the changes during the fermentation of the wine prepared from palm sap. Also, this study allowed for an exploration of the distinctive characteristics of indigenous *toddy* prepared from specific species of palm.

## 2 Materials and methods

### 2.1 Palm sap collection

The palm sap was extracted from ‘Wild Date Palm’ (*Phoenix sylvestris*) trees in the months of August–September from Simarbana (26.6235° N, 87.2768° E), situated in Itahari sub-metropolitan, Nepal. The location is characterized by a warm and temperate atmosphere. It has an elevation of 110 m above mean sea level (MSL). The mean annual temperature and annual precipitation recorded were 24.6 °C and 1891 mm, respectively. The mean temperature and the total precipitation in the month of sap collection were 26.7 °C and 383 mm, respectively.

### 2.2 Outline of the study

This study consisted of two distinct phases: fieldwork and laboratory work. First, the local farmers assisted in identifying the age of the palm trees during the fieldwork. The head of the stem was thinly sliced using a sanitized stainless-steel knife, and the lower angular tip of the resulting "V"-shaped cut was then filled with a hollow circular bamboo pipe and the opposite end of the bamboo pipe, was inserted into a pot. The sap was collected in a cleaned and rinsed stainless-steel pot and was gathered early the next morning. After that, the samples were sent to the laboratory after being pasteurized for 25 min at 75 °C.

Three age categories of palm trees were identified in order to determine the ideal age to yield high-quality palm sap: young (less than 15 years old), middle-aged (between 15 and 40 years old), and old (more than 40 years old). Two important criteria for classifying the tree were its total soluble solids (TSS) content and sap yield. The TSS and sap yield were recorded at 3PM, 9PM, 6 a.m., 9AM, and 3PM the next day. To choose the best tree, the results obtained were statistically analyzed.

Sap was similarly extracted from a tree that was at the ideal age for sap extraction to conduct further laboratory work. The pasteurized samples were aseptically placed into six amber-colored bottles (each with a 650-mL capacity, in total 2 L for each culture). After that, table sugar was added to adjust TSS to 21.5 °Bx. Among the six amber-colored bottles of wine, three were inoculated with a pure culture of *Saccharomyces cerevisiae* (SC-22) at a rate of 0.3 g/L sap, and the other three with the same amount of native palm wine. The changes during fermentation were studied in these samples.

### 2.3 Analytical methods

The physico-chemical analysis of the samples was carried out every 2 days for 12 days. The analytical parameters were pH, titrable acidity, volatile acidity, reducing sugar, total soluble solids, alcohol content, ester content, and aldehyde content. The analysis was conducted on different triplicate samplings.

#### 2.3.1 pH and total titratable acidity measurement

The pH was measured using a pH meter from Hanna Instruments. For pH determination, the pH meter was first calibrated with buffers of pH 4 and 7. Then the pH electrode was dipped in the sufficient sample, and the reading was noted.

The total titratable acidity was determined as per [20]. The samples of wine were initially degassed by stirring and heating them to 90 °C to remove carbon dioxide. Approximately 100 mL of distilled water was taken in a beaker and titrated with standard solution sodium hydroxide (NaOH) 0.05 N, with continuous magnetic stirring until pH 8.2. Then, 50 mL of the degassed sample of wine was taken in a beaker and titrated with a standard sodium hydroxide solution until pH 8.2. The volume of NaOH required was noted, and titratable acidity was calculated as shown below. The total acidity is expressed as tartaric acid.

$$\text{Total}\;\text{acidity}\;\text{as}\;\text{tartaric}\;\text{acid}\;g\;\text{per}\;\text{litre}\;\text{of}\;\text{wine}=\frac{V\times 0.00375\times 1000}{V_{1}}$$

Where, V: volume of wine taken for estimation.

V_1_: volume of standard NaOH used for titration, in mL.

Note: 1 mL of 0.05 N NaOH is equivalent to 0.00375 g of tartaric acid.

#### 2.3.2 Total solid, total soluble solid, and total insoluble solid

The total solid content was determined as the residue after evaporation [17]. For this, 200 mL of sap was transferred into a dried, weighed glass bowl and evaporated in a water bath. Then it was kept in an air oven maintained at 100 °C for 2 h. It was then cooled and weighed. The process was continued until a constant weight was obtained. Total solid was calculated as follows:

$$\text{Total}\;\text{solid}\;\%\;\left(\frac{w}{v}\right)=\frac{W_{1}-W}{V}\times 100$$

Where, W1 = weight of glass bowl with dry residue, in g.

W = weight of an empty glass bowl, in g.

V = volume of sap taken for the estimation, in mL.

Total soluble solid was determined using a standard calibrated hand refractometer and expressed as degree brix (°Bx) or percentage. Total insoluble solid was calculated by the difference method using the following formula:

Total insoluble solid= (total solid) – (total soluble solid).

#### 2.3.3 Volatile acidity measurement

The measurement of volatile acidity was done according to Ref. [20]. To determine volatile acidity, 100 mL of palm wine was diluted with 50 mL of distilled water, and distillation was performed in a laboratory distillation setup to collect the distillate. Then 50 mL of distillate was taken and titrated against 0.05 N sodium hydroxide (NaOH) using phenolphthalein as an indicator. Volatile acidity as an acetic acid gram per litre of palm wine was calculated using the formula:

$$\text{Volatile}\;\text{acidity}\;\text{as}\;\text{acetic}\;\text{acid}\;\left(g\;\text{per}\;\text{litre}\;\text{of}\;\text{palm}\;\text{wine}\right)=\frac{V\times 0.003\times 1000}{V_{1}}$$

Where, V1 = Volume of wine taken for estimation.

V = volume of standard NaOH used for titration, in ml.

Note: 1 mL of 0.05 N NaOH is equivalent to 0.003 g of acetic acid.

#### 2.3.4 Reducing sugar, non-reducing sugar and total sugar determination

The percentage of reducing sugar in palm wine samples was determined according to Kirk and Sawyer [21], with a slight modification. To standardize Fehling solution, 5 mL each of Fehling solution A and B were pipetted into a conical flask. Then, 15 mL of distilled water was added, and the mixture was boiled over a Bunsen flame for approximately 2 min. The next 3 drops of methylene blue were added, and the hot liquid was titrated with a standard dextrose solution (2.5 mg/mL) until the endpoint was reached, as indicated by the disappearance of the blue color of the indicator and the appearance of the reddish color of cuprous oxide precipitates. The Fehling factor was calculated using the following formula:

$$\text{Fehling}\;\text{factor}=\frac{\text{Titer}\times \text{mg}\;\text{reducing}\;\text{sugar}/\text{mL}}{1000}g\;\text{per}\;10\;\text{mL}\;\text{Fehling}$$

For the determination of reducing sugar content in the wine sample, 25 mL of sample was taken and neutralized with dilute NaOH. The volume was then adjusted to 100 mL with distilled water, and the solution was titrated with Fehling solution using a similar procedure as in the standardization of Fehling solution. The percentage of reducing sugar was calculated using the formula:

$$\%\text{Reducing}\;\text{sugar}=\frac{\text{Fehling}\;\text{factor}\times \text{dilution}\times 100}{\text{Aliquot}\;\text{titer}\times \text{volume}\;\text{of}\;\text{sample}\;\text{taken}}$$

For the determination of total sugar content, 25 mL of sample was taken in a conical flask, and 5 mL of concentrated HCl was added. The mixture was then heated in a water bath and boiled for 1 h. Afterward, the content was neutralized with NaOH, and the percentage sugar was determined as described for the reducing sugar determination. The non-reducing sugar was determined by subtracting the reducing sugar from the total sugar.

#### 2.3.5 Determination of alcohol content from a specific gravity method

The determination of the percentage of alcohol by volume from speciﬁc gravity was done as described in AOAC [22]. Initially, 100 mL of palm wine was diluted with 50 mL of water and then distilled in a laboratory setup. After collecting 100 mL of distillate, the relative speciﬁc gravity was determined. This was calculated by dividing the weight of 25 mL of the distillate by the weight of an equal volume of water using a 25-mL speciﬁc gravity bottle. The obtained value was then compared to a reference table to determine the percentage of alcohol by volume.

#### 2.3.6 Ester content

The total ester content was determined as described in Ref. [20]. In brief, 10 mL of 0.1 N sodium hydroxide (NaOH) was added to the neutralized distillate from the volatile acidity determination and refluxed on a steam bath for 1 h. Then, it was cooled, and the unspent alkali was titrated against standard 0.1 N sulphuric acid (H2SO4). Similarly, blank was carried out in the same way, simultaneously taking 50 mL of distilled water instead of distillate. The difference in titer value of standard sulphuric acid was calculated, and the ester content was calculated.

$$\text{Ester}\;\text{content}\;\text{as}\;\text{ethyl}\;\text{acetate}\;\left(g\;\text{per}\;100\;\text{litre}\;\text{absolute}\;\text{alcohol}\right)=\frac{V\times 0.0088\times 100\times 1000\times 2}{V_{1}}$$

Where, V = difference of titer value of standard H2SO4 used for blank and sample, in mL.

V1 = alcohol % by volume.

Note: 1 mL of 0.1 N NaOH is equivalent to 0.0088 g of ethyl acetate.

#### 2.3.7 Total aldehyde content

Total aldehyde expressed as grams of acetaldehyde per 100 L of alcohol was determined as per [20]. At first, distillate was prepared as described in Section 2.2.5. In a 250-mL iodine flask, 50 mL of distillate of liquor was taken, and 10 mL of sodium bisulphite (0.05 N) solution was added. The flask was kept for 30 min in a dark place with occasional shaking. A standard iodine solution of 25 mL was added to the flask and excess iodine was back-titrated against a standard sodium thiosulphate (0.05 N) solution using a starch (1 %) indicator to light green end point. Similarly, blank was carried out with 50 mL of distilled water. Then, the difference in titer value in mililiters of sodium thiosulphate solution was noted, and the aldehyde content was calculated.

$$\text{Aldehyde}\;\text{content}\;\text{as}\;\text{acetaldehyde}\;\left(g\;\text{per}\;100\;\text{litres}\;\text{of}\;\text{absolute}\;\text{alcohol}\right)=\frac{V\times 0.0011\times 100\times 1000\times 2}{V_{1}}$$

Where, V1 = alcohol % by volume.

V = difference in titer of blank and sample in ml of sodium thiosulphate solution.

Note: 1 mL of 0.05 N sodium thiosulphate is equivalent to 0.0011 g of acetaldehyde.

### 2.4 Sensory analysis

The sensory evaluation of final palm wines (both naturally fermented and pure-cultured) was conducted. Based on their prior experience and ability to distinguish the sensory characteristics (color, aroma, taste, and overall acceptability), fifteen skilled panelists were selected from the Central Department of Food Technology (CDFT). Sensory analysis was conducted in two different stages: a 9-point hedonic rating and an acceptability test. In the 9-point hedonic rating, panelists were instructed to score the samples within the range of 1–9 (1 = strong dislike and 9 = strong liking) based on their personal preferences of the individual sensory parameters, i.e., color, aroma, taste, and overall acceptance. For the acceptability test, the panelists evaluated the acceptability of the palm wine as described by Pimentel, Gomes da Cruz [23]. The indicators used for the acceptability test of color were clear, dull or cloudy, bright, dark, and pale light. While the indicators used for the acceptability test of aroma were fruity, powerful, subtle, putrid, and floral, in terms of taste, the indicators used were sweet, bitter, sour, salty, and dry.

Sensory evaluation was carried out in a clean, odor-free, and well-ventilated room. Clean and transparent glass beakers were used for serving. Panelists were first requested to take a rest for about 10 min prior to the sensory evaluation. Next, they were provided with 100 mL of palm wine and asked to provide a score based on their individual perceptions of the sensory parameters. Clean, potable water was made available for the panelists to rinse their mouths in between the testing of samples.

### 2.5 Statistical analysis

The statistical software IBMSPSS V-20 was used to analyze the results after the analyses were performed in triplicate. An analysis of variance (ANOVA) was used at a 5 % level of significance. Additionally, MS Excel 2016 was used for the preparation of graphs. The obtained data were presented as the mean value ± standard deviation (SD).

## 3 Results and discussion

### 3.1 Result of the survey conducted

A questionnaire (Appendix 1) survey was created to collect data on a number of topics related to palm wine (*toddy*), such as sap collection methods, utilization, market value, potential hazards, and more. The survey was carried out at a number of locations throughout the districts of Sunsari and Morang. Participants were chosen from among respondents who were knowledgeable about the collection, consumption, and marketing of palm wine. The study had 30 respondents in total, of whom 5 were collectors of palm sap, 15 were consumers, and 10 were small vendors or local marketers (retailers). There are two particular seasons for sap collection: Bhadra to Mangsir (September to December) and Falgun to Ashad (March to July).

According to the survey results, the people who collected palm sap were all men who had been doing this since they were young. These sap collectors ranged in age from thirty to fifty years. Because of the unstable market circumstances and relatively low earning potential of this family business, younger people—especially young men—showed less enthusiasm for pursuing it. The art of collecting palm sap was passed on to all of the respondents by their parents and grandparents.

These collectors tapped palm sap almost year-round and marketed their product. It revealed that the survey participants knew very little about the specific ages of palm trees and the most productive age range. But they were sure that the tree was at least 8–10 years old when it was tapped for the first time and that it would usually be tapped for at least 35–40 years. The collectors often tap the same tree for 4–5 days straight before letting it rest for another 4–5 days. This method relies on the belief that allowing the tree enough time to rest produces sap of higher quality. According to the respondents, tapping is not recommended during the summer or rainy seasons because of high rainfall and the shortened storage life of sap during these periods.

From the survey, 12 out of the 15 consumers that participated in the survey were found to be male. Furthermore, 85 % of the consumers were between the ages of 30–50, while the remaining 15 % were between the ages of 20–30. Out of the 15 customers, only 4 said they regularly drank palm sap or wine; the other 11 said they did it sometimes. The majority of consumers had good opinions about palm sap, highlighting its refreshing qualities, appealing taste, and satiety value as the main reasons that make it a favored beverage among those who enjoy it.

Ten small marketers or vendors, including 60 % males and the remaining 40 % females, were taken for the survey. 40 % of the vendors were regular marketers of palm sap or wine, i.e., selling the product every day, whereas 60 % were occasional marketers, i.e., only on specific market days.

### 3.2 Selection of best aged palm tree

To determine the best age for tapping palm trees, the tapping process was initiated at 3 p.m., and samples were collected at 9 p.m., 6 a.m., 9 a.m., and 3 p.m. the next day. The mean TSS content of the saps collected significantly differed from each other as shown in Table 1. Statistical analysis showed that collecting palm sap immediately from the tree is best in terms of TSS, but it was necessary to have a sufficient quantity of sap for the collection. It was observed that there was a significant reduction of TSS in the sap during the collection. For young tree, it was reduced from 14.13 °Bx to 3.53 °Bx; for middle-aged tree, it was reduced from 16.67 °Bx to 6.8 °Bx at the end of 24 h of tapping. For the old tree, it was found to be reduced from 14 °Bx to 9 °Bx. During the study, it was noted that the rate of TSS depletion was much higher during the day than at night. It was reported that there was no significant amount of sap that could be collected during the daytime, especially after sunrise. From the obtained result, it could be suggested that the ideal age of palm tree for sap collection is middle-aged palm tree, and the collection time is early in the morning at 6 a.m. Halim, Chowdhury [24] reported similar finding on *khajur* palm (*Phoenix sylvestris* Robx) of 7–14 years old yielding 2500 mL of sap on average each night and recommended the sap be collected early in the morning.

**Table 1:** TSS content, relative humidity and temperature of sap collected from different aged tree.

Duration (hr)	Temperature	RH	TSS content (^o^Bx)		
			Young aged	Middle aged	Old aged
0 (3 p.m.)	31.1	70 %	14.13 (0.30)^a^	16.67 (0.47)^b^	14 (0.34)^a^
6 (9 p.m.)	26.9	74 %	11.4 (0.20)^a^	14.67 (0.11)^b^	12.53 (0.11)^a^
15 (6 a.m.)	28.2	79 %	6.67 (0.11)^a^	12.47 (0.11)^b^	11.53 (0.11)^b^
18 (9 a.m.)	32.2	77 %	5.067 (0.30)^a^	9.07 (0.23)^b^	10.13 (0.11)^c^
24 (3 p.m.)	34.7	75 %	3.53 (0.11)^a^	6.8 (0.20)^b^	9 (0)^c^

*Values are mean of triplicate and values in the parentheses are the standard deviation of the triplicate values. Figures in the row bearing different alphabet in superscript are significantly different at p ≤ 0.05.

### 3.3 General composition of palm sap

The fresh pasteurized palm sap was taken, and the results obtained after analysis are presented in Table 2. Naknaen and Meenune [25] reported similar findings when they analyzed pasteurized sap from different regions of Thailand. Ideally, the alcohol content of fresh palm sap should be zero. However, the alcohol content of fresh palm sap was found to be 0.86 %, which may be due to the fermentation that took place during sap collection. According to Oluwole, Kosoko [24], the *neera* (traditional name for palm sap) contains 13–14 % total soluble solids, a pH below 6, and an alcohol content of up to 2.32 % if the sap is collected traditionally. The general composition of the palm sap was within the range reported by Oluwole, Kosoko [26].

**Table 2:** Analysis of fresh palm sap selected for the fermentation.

Parameter	Values
pH	4.9 ± 0
**Solid content (% wet basis)**	
Total solid (TS)	14.57 ± 0.12
Total soluble solid (TSS)	11.9 ± 0.14
Total insoluble solid	2.71 ± 0.08
**Acidity (g/100 mL sap)**	
Total acidity (as tartaric acid)	1.22 ± 0.04
Volatile acidity (as acetic acid)	0.03 ± 0
**Sugar (g/100 mL sap)**	
Total sugar (as dextrose)	10.08 ± 1.06
Reducing sugar (as dextrose)	0.54 ± 0.03
Non-reducing sugar (as sucrose)	9.21 ± 0.62
Specific gravity of distillate at 26 °C	0.99 ± 0.0003
Alcohol by volume (%)	0.86 ± 0

The study was conducted over a period of 12 days to observe changes in both the physico-chemical and distillate properties of the fermented palm sap. The study continued until there were no significant changes observed in the total soluble solids (TSS) and pH. The physico-chemical properties included total soluble solids (TSS), pH, reducing sugar, total acidity, and volatile acidity, whereas the distillate properties include specific gravity, alcohol content, aldehyde content, and ester content.

#### 3.3.1 Changes in physico-chemical properties of palm wine

The physico-chemical properties of pure culture fermented and naturally fermented palm wines showed significant changes (p ≤ 0.05) throughout the fermentation process.

##### 3.3.1.1 Changes in total soluble solid (TSS)

**Fig. 1:**
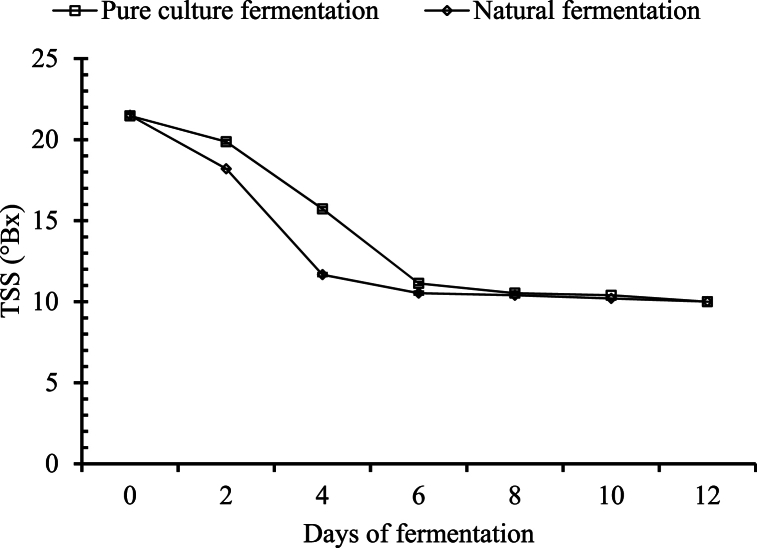
Changes in TSS during the course of fermentation.

Total soluble solids (TSS) of the palm wines were recorded for 12 days, and there was a significant decrease in total soluble solids in this period of 12 days. The pattern of TSS depletion is shown in Fig. 1. There was a significant amount of change in the total soluble solids (p ≤ 0.05) along with the increase in the number of days. TSS was measured every 2 days up to the 12th day for both palm wines. In the pure cultured palm wine, a significant drop in total soluble solids was observed along with the increase in days, whereas there was no significant change in TSS in the naturally fermented palm wine from day 6 to day 8, day 8 to day 10, and day 10 to day 12.

The initial TSS of pure culture fermented and naturally fermented palm wines were 21.47 and 21.5°Bx, respectively. The depletion of sugar was at a high rate for the first 4 days of natural fermentation (up to 11.67 %), whereas for pure cultured fermentation it was at a high rate for 6 days (11.13 %). After that, the rate of decrease in sugar was slow and steady for both fermentation systems. When TSS was reduced to 10 °Bx, it was observed that there was no significant depletion of sugar for the following days.

Hebbar, Pandiselvam [27] reported that the TSS of sap decreased up to 6 % from 15 %. Similarly, Xia, Li [28] kept coconut inflorescence sap under natural fermentation for 11 days and found a reduction of total sugar from 15 % to 6 %. Nooralabettu and Valder [29] also reported a similar pattern of reduction in sugar level during the fermentation of *Borassus flabellifer* sap using the isolates of *Saccharomyces cerevisiae*.

##### 3.3.1.2 Changes in pH

**Fig. 2:**
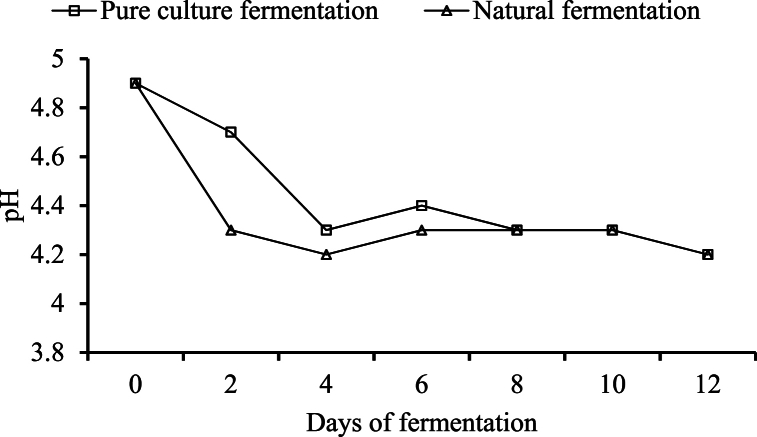
Changes in pH during the course of fermentation.

During the study, it was observed that there was a sharp drop in pH until day 4 for pure culture and it was until day 2 for the natural fermentation system. After 12 days, there was no significant change in pH (p ≤ 0.05). The changes in pH are shown in Fig. 2.

Hebbar, Arivalagan [30] observed a correlation between the sugar content of sap and pH during fermentation. Fresh sap, when stored at ambient conditions, initially undergoes lactic acid fermentation, followed by alcoholic fermentation, and finally acetic acid fermentation due to the influence of the mixed microorganisms. As the sap ferments, it becomes acidic, leading to a reduction in pH. The freshly collected sap starts fermenting within 2–3 h at ambient temperature, and the pH starts to decline. Hebbar, Pandiselvam [27] reported that the pH of palm sap can drop up to 3.5 from its nearly neutral pH. Danmadami, Yabaya [31] also observed a decrease in pH from 6.2 to 3.8 within 24 h of the initiation of the fermentation process in *burukutu* brewed using *S. cerevisiae* isolated from palm wine. Karamoko, Moroh [32] reported that the pH of *dura* and *tenera*, species of *Elaeis guineensis*, lowered from 5.23 to 3.44 and 4.64 to 3.43, respectively, after 28 days of sap tapping. The decline of pH is associated with an increase in total acidity [13,14].

##### 3.3.1.3 Changes in reducing sugar

**Fig. 3:**
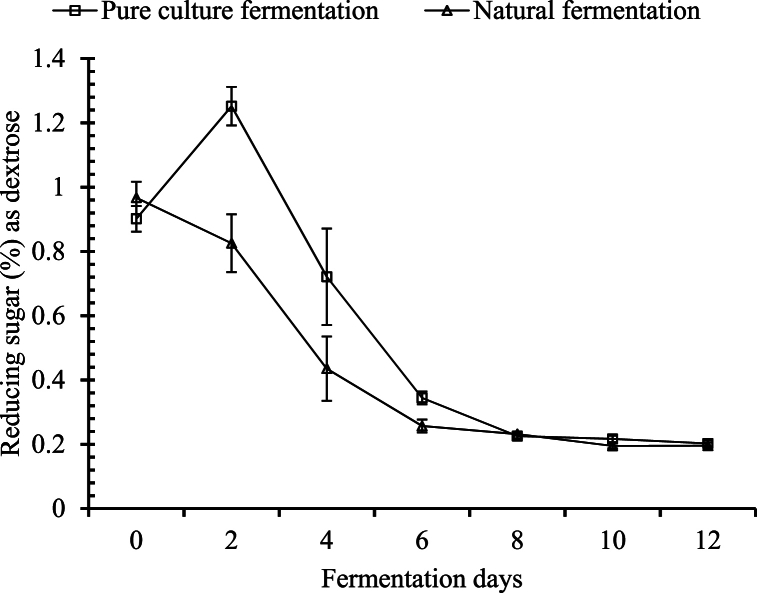
Changes in reducing sugar during the course of fermentation.

For pure culture fermentation, there was a slight increase in reducing sugar in the first 2 days of fermentation, although it was not significant. In the case of the naturally fermented sample, there was a significant change in reducing sugar noticed only on the 4th day of fermentation, as shown in Fig. 3. After the 4th day, there was no significant change in reducing sugar in the naturally fermented samples. Xia, Li [28] observed an increase in reducing sugar during the initial 3 days of fermentation, and thereafter it was decreasing. They suggested that the initial increase in the level of reducing sugar was due to the inversion of sucrose. After that, the reduction observed was due to the microbial consumption of sugar. In this study, an initial increase followed by a decreasing pattern for the pure cultured system was observed. In case of the natural fermentation system, it was decreasing from the initial. It might be because the microbial consumption rate of reducing sugar is constantly higher than the rate of inversion. Kapilan [16] also reported a gradual decrease in reducing sugar in the natural fermentation of *Caryota* sap with inhibitory substances as well as in the control group.

##### 3.3.1.4 Changes in total acidity

**Fig. 4:**
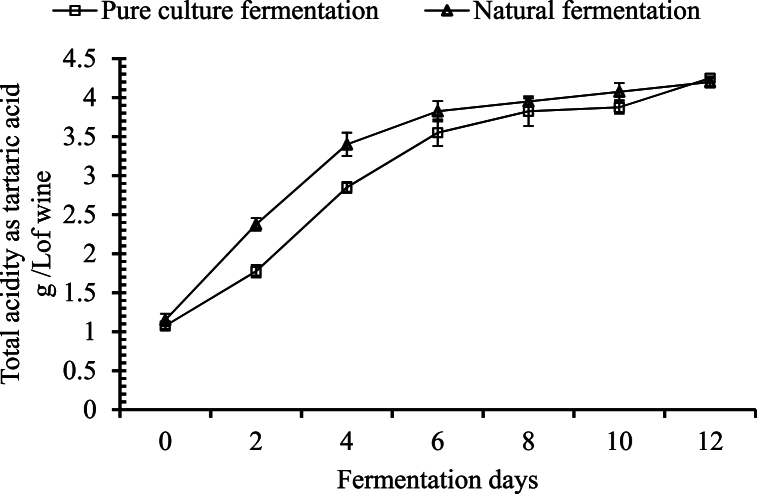
Changes in total acidity during the course of fermentation.

With the reduction in pH level, a reciprocal increase in the amount of total acidity (as tartaric acid %) was observed as the fermentation progressed. The amount of total acidity increased with a significant difference (p ≤ 0.05) from day 0 to day 12. A significant difference was observed in the palm wine fermented with pure culture in each interval of 2 days, except from day 8 to day 10. In the case of the naturally fermented palm wine, the total acidity was found to be significantly similar from day 6 to day 8, day 8 to day 10, and day 10 to day 12 (Fig. 4).

There was a gradual increase in the total titratable acidity of both pure culture and natural fermentation systems. A similar result was reported by Danmadami, Yabaya [31], when *burukutu* was prepared using starter culture and *Saccharomyces cerevisiae* isolated from palm wine. However, the rate of increase in titratable acidity was faster in experimental palm wine with starter culture than in that with pure culture of *Saccharomyces cerevisiae*. The increase in titratable acidity leads to a decrease in pH level as the fermentation time increases. This suggests that using only *Saccharomyces cerevisiae* in palm sap fermentation to produce palm wine consumes the sugar at a steady rate and produces a limited amount of acids, which can enhance the flavor compared to wine produced using a starter culture [28]. The total acid content increased constantly from day 1 to day 4, which is likely to be in the lactic acid fermentation. This increased slowly from day 4 to day 6 until the sap began its ethanol fermentation. This condition might therefore enhance the growth and invertase activity of the yeasts [33]. These changes in the total acid do not completely, however agree with the results of Atputharajah, Widanapathirana [33], where the total acid content increased from day 1 to day 5 but slowly decreased afterward. The difference might be due to the variations in microorganisms in the sap. Karamoko, Moroh [32] showed an increase in total acidity in palm wine from palm tree *Elaeis guineensis* of two varieties namely *dura* and *tenera* during the course of 28 days of tapping.

##### 3.3.1.5 Changes in volatile acidity

**Fig. 5:**
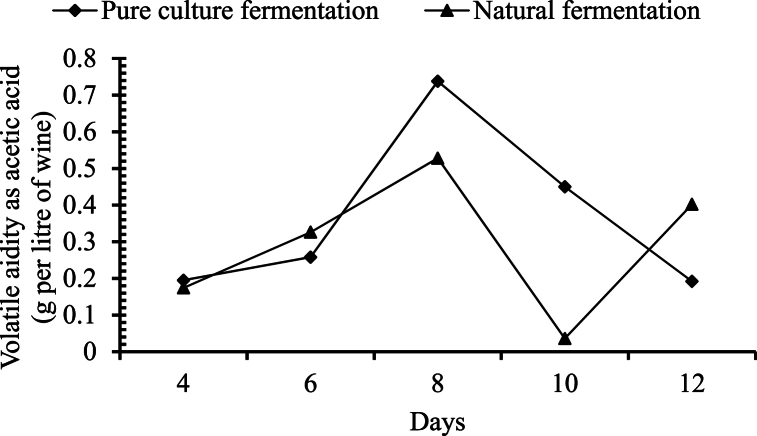
Changes in volatile acidity during the course of fermentation.

Volatile acidity was found to be increased with the time for 8th days of fermentation for both the systems. For the fermentation with pure yeast culture, volatile acidity was decreased significantly after 8th day of fermentation. But in case of natural fermentation, volatile acidity was found to be decreased from 8th day to 10th day of fermentation which was again significantly increased to 12th day of fermentation (Fig. 5).

In palm wine, volatile acid primarily consists of acetic acid. The rapid increase in volatile acid was observed only on day 4 when compared with total acids. This suggests that the acetic acid fermentation starts after day 4 and the decrease in volatile acid after day 8 is due to the conversion of acid into esters and aldehydes along with the formation of flavoring components [28]. Atputharajah, Widanapathirana [33] reported no changes in volatile acid of fermented sap prepared from coconut palm. Moreover, the volatile acid content showed a slight increase from day 1 to day 5, and it increased sharply thereafter, as shown in Fig. 5. This pattern showed that the process was in the acetic acid fermentation phase after day 5.

#### 3.3.2 Changes in volatile constituents of palm wine

The kinetics of volatile constituents present in palm wine were analyzed only after day 4. Alcohol content, ester content, and aldehyde contents were analyzed from day 4 to day 12 at a 2-day interval.

##### 3.3.2.1 Changes in alcohol content

**Fig. 6:**
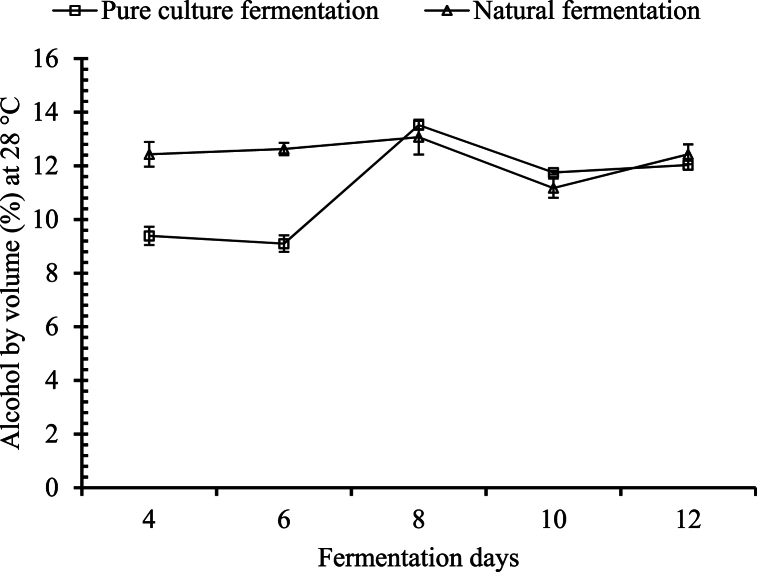
Changes in alcohol content during the course of fermentation.

The total alcohol by volume (ABV) was calculated in the temperature range of 28–30 °C starting from day 4. A significant difference (p ≤ 0.05) in the total amount of alcohol by volume was observed as days progressed for both palm wines. In the palm wine fermented with the pure culture, no significant change was observed from day 4 to day 6. However, there was a significant increase from day 6 to day 8 and from day 8 to day 10, and it remained significantly similar from day 10 to day 12. In the case of the naturally fermented palm wine, the amount of alcohol by volume remained significantly similar for days 4, 6, 8, and 12. The wine on the 10th day had a significantly lower alcohol content (Fig. 6).

The production of alcohol was at a high rate for the initial 8 days of fermentation, after which it decreased. The rate of alcohol production was higher in the case of natural fermentation than in pure culture fermentation. Natural microflora contains several yeasts other than *Saccharomyces cerevisiae*, such as *Saccharomyces chevalieri* and *Zymomonas mobilis*, which are responsible for alcoholic fermentation [10]. The alcohol content was at its maximum (i.e., 13 %) at day 8 for both systems, and later it decreased to 12 %. Xia, Li [28] reported higher production of alcohol on day 7 (i.e., 9 %), and later it decreased to about 8 % on the 11th day of fermentation. Hebbar, Pandiselvam [27] reported a similar pattern of alcohol production in palm wine. Atputharajah, Widanapathirana [33] suggested that a decline in alcohol level at later stages of fermentation can result from acetic acid fermentation by yeast and bacteria present in palm. Such a decline may also be due to the conversion of alcohol to aldehydes, esters, and other volatile compounds. Karamoko, Moroh [32] reported that ethanol content can be increased up to 3.18 and 2.5 in the case of *dura* and *tenera*, respectively, after 28 days of tapping if additional sugars are not allowed. Generally, *toddy* contains less than 6 % ABV, which make it low- or mild-alcohol containing beverage [13]. But in this case, due to the additional sugar, the alcohol content was higher than that of the naturally fermented palm wine.

##### 3.3.2.2 Changes in ester content

**Fig. 7:**
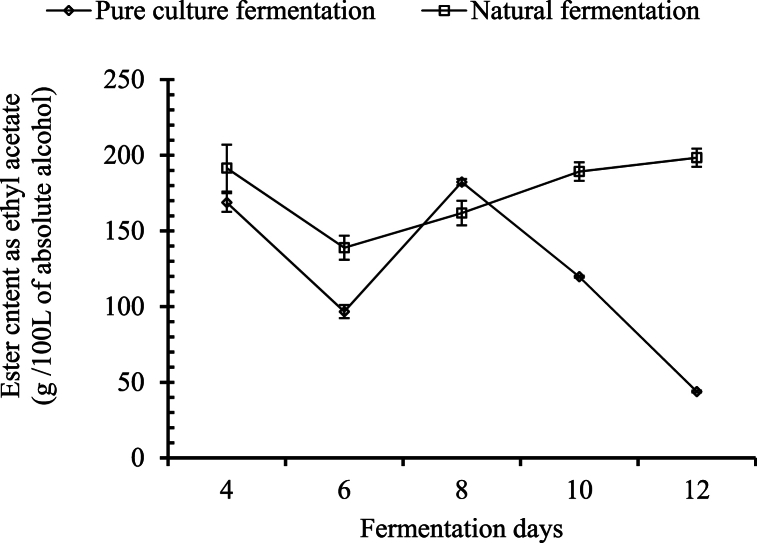
Changes in ester content during the course of fermentation.

The total amount of ester was calculated in terms of ethyl acetate per 100 L of absolute alcohol, starting from day 4 for both systems of palm wines. In both palm wines, a significant difference (p ≤ 0.05) was observed in the amount of ester with the change in number of days. In the palm wine using pure culture, the total amount of ester decreased significantly after the fermentation was completed, and a significant difference was also observed in each 2-day interval (Fig. 7). In the palm wine fermented with natural culture, the amount of ester was significantly similar on days 4, 10, and 12, whereas the ester content on days 6 and 8 was significantly different from the other days. A similar result was observed by Uzochukwu, Balogh [34].

Nwaiwu, Ibekwe [17] reported that the total ester content during active or mid-fermentation was 7.3 times higher than in the early stages of fermentation. In actively fermenting palm wine, it constituted about 30.48 % of the volatiles. Most esters detected had been reported in the headspace of palm wine. Uzochukwu, Balogh [34] reported that the primary esters present in palm wine included acetate esters of straight-chain alcohols and ethyl esters of straight-chain carboxylic acids. Esters are produced through microbial action by combining acids and alcohol via the esterification reaction in different alcoholic beverages, such as *baijiu* from China [13,35]. Acetic acid and ethanol can combine to form ethyl acetate [36]. This could possibly cause a reduction of ethanol and acetic acid, resulting in an increase in the intensity of the ethyl acetate aroma.

##### 3.3.2.3 Changes in aldehyde content

**Fig. 8:**
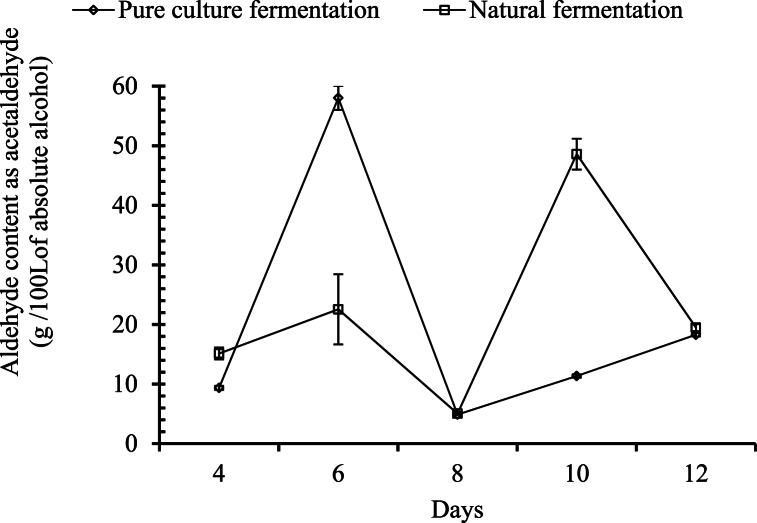
Changes in aldehyde content during the course of fermentation.

A significant difference (p ≤ 0.05) in the aldehyde content was observed as the number of days increased in both palm wines. In the palm wine fermented with the pure culture, the amount of aldehyde increased significantly from day 4 to day 6, followed by a significant decrease from day 6 to day 8, and there was another significant increase in aldehyde content from day 8–12, as shown in Fig. 8. In the case of the palm wine without the pure culture, the aldehyde content increased significantly from day 4–6, followed by a significant decrease from day 6 to day 8. This was again followed by a significant increment in aldehyde content from day 8 to day 10, but then a significant drop occurred in the aldehyde content on day 12. A similar pattern of result was observed in the natural fermentation of palm sap by Uzochukwu, Balogh [34]. Acetaldehyde concentration peaks during the initial stage of alcoholic fermentation, primarily due to yeast and enzymatic metabolisms. Both spontaneous and selected *Sachharomyces cerevisiae* yeasts release acetaldehyde early in the fermentation process. After this peak, the acetaldehyde levels decrease as it gets partially re-utilized. Production levels vary significantly depending on fermentation conditions and the predominant yeast species involved. In addition to *Sachharomyces cerevisiae*, other yeast species, such as *Schizosaccharomyces pombe* and *Zygosaccharomyces bailii*, also contribute to acetaldehyde production in wine [37,38]. In the palm wine of *Elaeis guineensis*,a total of 11 carbonyl compounds have been identified, with acetaldehyde being one of the most abundant carbonyls in palm wine [34]. Borse, Rao [39] reported the highest production of carbonyl compounds up to 2249.65 μg/L of wine with 5.17 % alcohol by volume (ABV), which is equivalent to 43.51 g/100 L of absolute alcohol. The production of aldehyde and other carbonyl compounds is one of the biochemical products of fermentation, and it occurs at the expense of alcohol. Ohimain [40], El-Maghrabey, El-Shaheny [41] reported that the conversion of alcohol, especially methanol and ethanol, to respective aldehydes is normal in the alcoholic fermentation process by alcohol dehydrogenase enzymes.

### 3.4 Sensory analysis

**Fig. 9:**
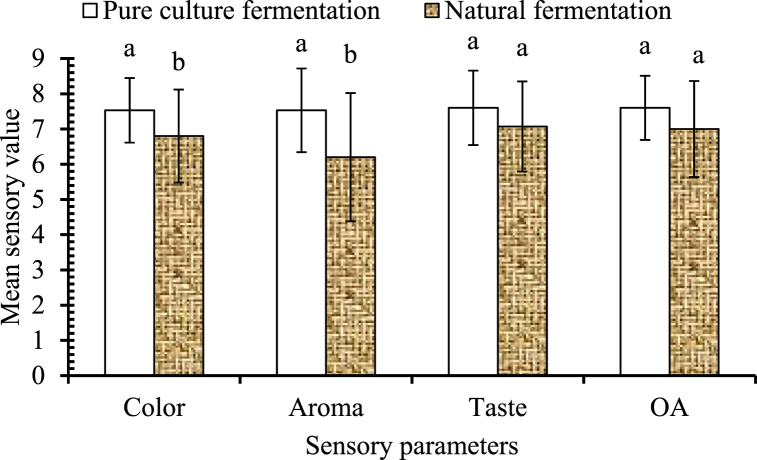
Mean sensory score for color, aroma, taste and overall acceptance (OA) of the samples. * Different alphabets on the top of the bars showed significant difference (p ≤ 0.05).

Panelists were instructed to select a single response parameter that is most preferred or noticeable to them in the acceptability test and also to provide a score in the 9-point hedonic rating. Fig. 9 illustrates the mean sensory scores provided by the panelists. For the acceptability test, clear, cloudy, bright, dark, pale, and light were taken as responses. Fruity, powerful, subtle, putrid, and floral were taken as response parameters for aroma, and sweet, bitter, sour, salty, and dry were taken as response parameters for taste. The acceptability of sensory parameters by the panelists is shown in Table 3.

**Table 3:** Acceptability test table for color, aroma and taste of samples.

Color	No. of respondent			No. of respondent			No. of respondent	
	PCF	NF	Aroma	PCF	NF	Taste	PCF	NF
Clear	2 (13.3 %)	1 (6.7 %)	Fruity	7 (46.7 %)	4 (26.7 %)	Sweet	11 (84.6 %)	9 (69.2 %)
Cloudy	11 (73.3)	14 (93.3 %)	Powerful	3 (20 %)	3 (20 %)	Bitter	1 (7.7 %)	3 (23.1 %)
Bright	0	0	Subtle	2 (13.3 %)	2 (13.3 %)	Sour	1 (7.7 %)	1 (7.7 %)
Dark	0	0	Putrid	0	2 (13.3 %)	Salty	0	0
Pale	1 (6.7 %)	0	Floral	1 (6.7 %)	0	Dry	0	0
Light	1 (6.7 %)	0	Other	2 (13.3 %)	4 (26.7 %)	Other	0	0
Other	0	0						
Total	15 (100 %)	15 (100 %)		15 (100 %)	15 (100 %)		13 (100 %)	13 (100 %)

Note- PCF: Pure culture fermentation and NF: Natural fermentation system.

#### 3.4.1 Color

The mean sensory score for color was 7.53 for the pure cultured sample and 6.8 for the natural fermentation sample. There was a significant difference (p ≤ 0.05) in color between the samples. From the hedonic sensory analysis and acceptability test, it was observed that a significant number of panelists preferred the pure-cultured sample over the mixed-cultured one. The color of the pure cultured samples was highly preferred by the panelists, whereas the mixed cultured samples received slightly lower ratings. This preference can be attributed to the acceptability test results, where most of the panelists noticed a cloudy color in the mixed-cultured sample when compared to the pure-cultured one. It showed that panelists were less inclined to like the cloudy color of the palm wine. A similar characterization of the palm wine was reported in several studies [8,18,33,[42], [43], [44]].

Naknean, Meenune [8] conducted an analysis of the turbidity of palm sap by measuring the transmittance value at 650 nm, revealing that turbidity levels ranged from 39.56 % to 79.95 %. In general, the immediate turbidity observed in fresh juice is primarily attributed to the presence of cell fragments. Additionally, haze formation can contribute to the turbidity of the juice. The turbidity of palm sap is greatly influenced by its protein concentration and the presence of polyphenol compounds. The interaction between proteins and polyphenols can lead to the formation of complexes, resulting in larger colloidal particles or haze, which may be responsible for the cloudy appearance of palm wine [45]. The formation of haze may result from interactions between proteins and sugars or metal ions [46]. Microorganisms are also responsible for the turbidity of palm sap. Uzochukwu, Balogh [47] suggested that the key microorganisms for the fermentation of palm sap are primarily *Saccharomyces* yeasts and lactic acid bacteria (LAB). Lactic acid bacteria are responsible for the consistency and soluble white coloration of palm sap due to the production of gum, likely dextrans, in the initial stage of fermentation. Furthermore, heavy suspensions of yeast and bacteria are also responsible for the milky-white appearance, resulting to an increase in the turbidity of palm sap. There was an absence of lactic acid bacteria in the pure cultured system, resulting in less whiteness in the sample.

#### 3.4.2 Aroma

The mean sensory score for aroma was 7.53 for the pure cultured sample and 6.2 for the natural fermentation sample. There was a significant difference between the samples, with the pure cultured sample being more preferred by the respondents. The “other type of aroma” refers to those aromas detected but not recognized by the panelists in the given list of responses. From the statistical analysis, it was concluded that for pure culture, a fruity aroma was most noticeable and was liked very much. Similarly, for the mixed-cultured system, panelists seem to be more confused by the aroma. For the natural fermentation sample, we can conclude that the aroma was fruity and powerful but was less liked by the panelists.

The chemical analysis revealed significantly lower levels of ethyl acetate in the pure cultured system compared to the natural fermentation system. Nwaiwu, Ibekwe [17] analyzed the principal aroma component of palm wine and reported that ethyl acetate had the most intense aroma. Ethanol and acetic acid can combine to form ethyl acetate, which possibly causes depletion of ethanol and acetic acid and probably results in an increase in ethyl acetate aroma intensity. Naknean, Meenune [8], and Shetty, D'Souza [18] have reported the involvement of various types of yeast and bacteria in the natural fermentation of palm sap to produce aromatic components. It is responsible for the higher concentration of aromatic components in a mixed-cultured system than in a pure-cultured system. Due to these microbes various types of desirable and undesirable aromatic components might be synthesized in the mixed culture fermentation while in case of natural fermentation variation might occur in less amount, which might be the reason for the less preference to aroma of mixed culture wine. Uzochukwu, Balogh [34] also found that ethyl esters in rose wines were correlated with the intensity of tree fruit aroma, while acetate esters were linked with tropical fruity notes. Therefore, it is reasonable to assume that these esters in palm wine contribute to its fruity bouquet. Lasekan, Buettner [48] reported that 41 odorant compounds were detected in palm wine (*Elaeis guineensis*), among them ethyl hexanoate and 3-methylbutyl acetate were reported to be responsible for the fruity note of palm wine.

#### 3.4.3 Taste

The mean sensory score for taste was 7.6 for the pure cultured sample and 7.07 for the mixed cultured sample. There was no significant difference between the samples in terms of taste. From the statistical analysis, it can be concluded that both samples were sweet in taste and were moderately liked by the panelists. The sweetness in the samples was because of the presence of residual sugar. Studies suggest that the taste of palm wine can vary considerably and depends on several factors, like the level of residual sugar, alcohol content, fermentation duration, and aromatic components [8]. In this study, residual sugar was the dominant factor for taste. Although there was no significant difference between the samples, the pure cultured sample was slightly more preferred by the respondents because of other factors like aromatic components, higher acid content, etc.

#### 3.4.4 Overall acceptance

The mean sensory score for overall acceptance was 7.6 for the pure cultured sample and 7 for the natural fermentation sample. Statistical analysis showed that there was no significant difference between the samples with respect to their taste and overall acceptance whereas color and aroma showed significant difference between the samples, although the pure-cultured sample was slightly more preferred by the respondents. This preference for the pure cultured sample may have been influenced by its attractive color and aroma. Danmadami, Yabaya [31] prepared *burukutu* with the pure culture of *Saccharomysis cerevisiae* isolated from palm wine and with starter culture and found that the *burukutu* produced from pure culture was more preferred by the respondent than with starter culture.

## 4 Conclusions, limitation and future prospect

The findings of the study concluded that during the fermentation process, significant variations were observed in the physico-chemical properties of palm wine as well as the volatile constituents in both pure culture and natural fermentation systems. In terms of acceptability, palm wine was characterized as having a cloudy appearance, a fruity aroma, and a sweet taste. With a methodological approach to control fermentation conditions and the selection of appropriate yeast strains, the quality and uniformity of palm wine can be improved, which can have an important impact on the wine industry. However, this work was limited to the sample collected from a single location, and the parameters studied were selective.

The food and beverage (F&B) sector is becoming more and more concerned with sustainability; therefore, improvement in extraction techniques for palm sap can have a positive impact on this sector. Palm sap wine has the ability to attract and reach a wider consumer base globally outside of its typical areas because of the increasing interest shown by consumers in cultural and traditional drinks.

## Data availability statement

The data related to the study are made available in the article.

## Ethics approval and consent to participate

The participants of survey and sensory evaluation voluntarily took part and scored the samples with full satisfaction. They were verbally informed about the study as we do not require any legal ethical consent requirements for the study.

## Funding statement

None.

## CRediT authorship contribution statement

**Nabin Khadka:** Validation, Software, Resources, Methodology, Investigation, Formal analysis, Data curation, Conceptualization. **Dev Raj Acharya:** Validation, Supervision, Resources, Methodology. **Anish Dangal:** Writing – review & editing, Writing – original draft, Visualization, Validation, Software. **Kishor Rai:** Writing – review & editing, Writing – original draft, Software. **Gaurav Gurung:** Software, Resources. **Girija Sherma:** Software, Resources. **Sabin Bahadur Khatri:** Software, Resources. **Navin Gautam:** Writing – review & editing, Software, Resources.

## Declaration of competing interest

The authors declare that they have no known competing financial interests or personal relationships that could have appeared to influence the work reported in this paper.
