# Supplementary material for: Study on the changes during the fermentation of the wine prepared from palm (Phoenix sylvestris) sap
Source: Heliyon. 2024 Aug 5;10(15):e35799. doi: 10.1016/j.heliyon.2024.e35799 (PMC11337016; doi:10.1016/j.heliyon.2024.e35799)
Supplement: Multimedia component 1 [file mmc1.docx]

**Questionnaire**

**General Questionnaire**

1. Name:

2. Location:

3. Age:

4. Occupation:

5. How long have you been harvesting toddy from palm tree?

6. Is this a family occupation (if yes, how many generation)?

7. Who do you usually get as your consumer?

8. What are the possible adulterations in final product?

9. What is the possible future for this product? What can be done to improve its status?

**Research questionnaire**

1. How old is the palm tree for its first harvest?
2. Till how old do keep harvesting the palm?
3. Which aged group trees give the most desirable palm sap?
4. Which is the most favourable time of tapping?
5. How long does it take for alcoholic fermentation to start?
6. What are the tools used for tapping?
7. How long is tapping done?
8. How long do you give rest to tree?
9. What is the expected amount of palm sap per tapping?
10. What is the desirable characteristics of good sap and toddy (color, flavor, aroma, taste)
